# Autologous Fat Transfer: An Aesthetic and Functional Refinement for Parotidectomy

**DOI:** 10.1155/2014/873453

**Published:** 2014-01-08

**Authors:** Pierre G. Vico, Axel Delange, Axel De Vooght

**Affiliations:** Department of Plastic Surgery, Clinique Saint Jean, 32 Boulevard du Jardin Botanique, Bte 29, 1000 Brussels, Belgium

## Abstract

Parotidectomy is a surgical procedure associated to functional (Frey's syndrome) as well as aesthetic (facial asymmetry) complications that can be very disturbing for the patient. Several procedures have been described to primarily avoid or secondarily reconstruct the facial defect and treat the neurological iatrogenic syndrome. Autologous fat transfer was primarily used in 10 cases to avoid such complications. It is an easy technique widely used in cosmetic and reconstructive surgery. This technique gives very satisfying long-term results on the cosmetic as well as on the physiological point of view.

## 1. Introduction

**Figure 1:**
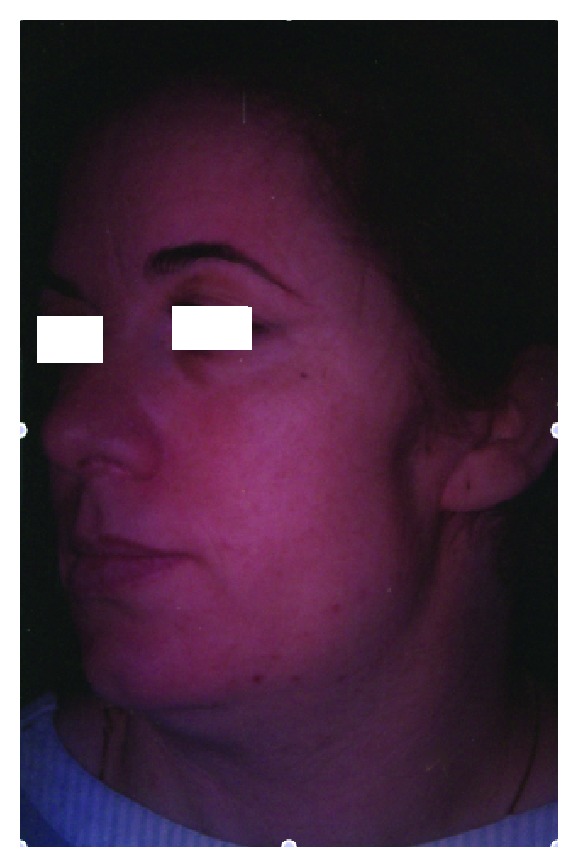
56 year-old patient, one year after a left parotidectomy without any primitive reconstructive procedure, presenting an obvious concave preauricular deformity. A secondary lipofilling was performed, leading to a permanent facial paresis and a poor cosmetic result.

Superficial or total parotidectomy is a common procedure associated with some possible complications as well as functional and aesthetic consequences. Among these, Frey's syndrome, also called gustatory sweat syndrome or auriculotemporal syndrome, and a preauricular deformity are common issues that can lead to very disturbing clinical situations. Indeed, Frey's syndrome is characterized by a sweating and a flushing and/or an erythema of the cheek area during mastication after parotid surgery. Aesthetic consequences include a concave facial effect with facial asymmetry (Figure 1) and prominent scar. Several procedures have been developed to primarily avoid or secondarily reconstruct the facial defect and treat the neurological iatrogenic syndrome.

Autologous fat transfer, also called fat graft or lipofilling following the operative technique used, is an easy technique widely used in cosmetic surgery to correct folds of the face (nasolabial folds, etc.) or to remodel soft tissue and enlarge or resurface them. This technique was primarily applied in 10 cases of superficial and total parotidectomy, with very good to excellent long-term results on the cosmetic as well as on the physiological point of view.

## 2. Material and Methods

Ten patients (4 males and 6 females) were operated on from January 2006 to December 2009 for clinically benign tumors of the superficial and/or deep lobe of the parotid gland. Dissection of the parotid was performed under magnification, with dissection and preservation of the facial nerve in all cases. Before closing the operative field, fat was harvested in the lower abdominal wall following Coleman's technique: after infiltration of the site of fat removal with a solution of 1% lidocaine with 1 : 200,000 epinephrine, a blunt tip cannula attached to a 10 cc Luer-Lok syringe is used to harvest fat. Care is taken to avoid mechanical trauma of the fatty tissue; the negative pressure is therefore limited to 5 cc in the barrel of the syringe. Contrary to Coleman, we do not centrifugate the harvested fat but we wash it with physiological salt solution. After washing, fat is put *en bloc*, in the operative field, at the place of the resected gland (mean: 13.6 mL, range: 4–20 mL). *En bloc* does here signify that fat was simply deposited in the operative defect and not injected in a multilayered way. A multilayered injection in any other preserved tissue (like in the masseter muscle, in the deep lobe of the parotid gland when preserved, or skin) was indeed not possible or not an option. Prevention of Frey's syndrome implies that fat should be interposed between skin and facial nerve.

This additional procedure is fast and performed in 5 to 10 additional minutes. This is not too time consuming, especially in case of a two-team approach. The mean operative total duration is 3 hours. A drain without suction is placed at the end of the procedure and a moderately compressive dressing is done. Drain and dressings are taken away on the 2nd postoperative day.

## 3. Results

Histology showed 3 well-encapsulated malignant tumors (one monomorph adenoma with foci of adenoid cystic carcinoma, one oncocytic carcinoma, and one adenoid cystic carcinoma). Those last patients were secondarily irradiated. All patients but one (loss of follow-up) are followed every 3 months the first year, and after that once a year, dependent on the compliance of each of them.

## 4. Case Reports

### 4.1. Case 1

**Figure 2:**
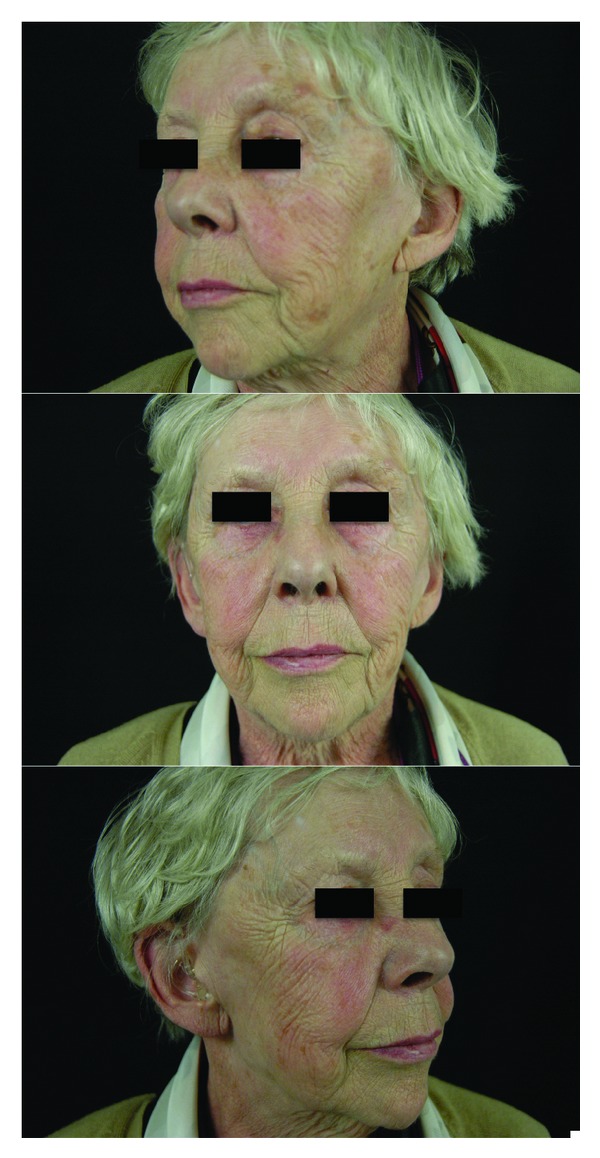
77 year-old woman 15 months after a right parotidectomy and an immediate autologous fat graft (18 mL of fat for 32 gr of parotid parenchyma resected). A Minor's starch-iodine test was performed 6 and 14 months after the procedure and remained negative.

This 77 year-old woman presents a tumor of the right parotid. Among the medical history, it should be noted that she has had a mastectomy for a breast cancer and a diabetes mellitus. The tumor is 3 cm in diameter, developed in the lower pole of the gland. A preoperative CT scan confirms the parotid location of the tumor and a fine needle biopsy under ultrasonographic control is in favor of a pleomorphic adenoma. She is operated under general anaesthesia, with magnification and neurostimulation of the facial nerve. The superficial lobe is excised, the facial nerve is preserved, and 18 mL of fat is put in the defect. Pathology confirms the preop diagnosis. She suffers no complications. A Minor's iodine-scratch test is performed 6 and 14 months after the procedure and remains negative. Follow-up is 15 months with a very good aesthetic result (Figure 2).

### 4.2. Case 2

**Figure 3:**
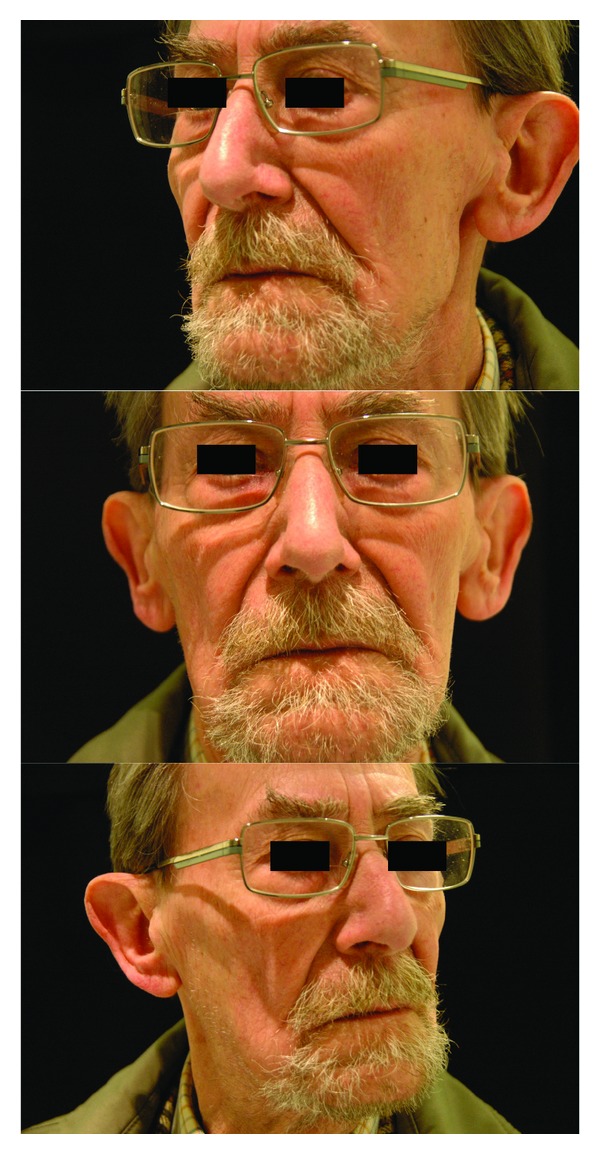
This 85 year-old male underwent a total left parotidectomy with dissection and preservation of the facial nerve and a fat graft (10 mL of fat for 51 gr resected). Follow-up at 46 months shows a very good cosmetic result and no complain of gustatory sweat syndrome is experienced by the patient. At one year, the Minor's starch-iodine test is negative.

**Figure 4:**
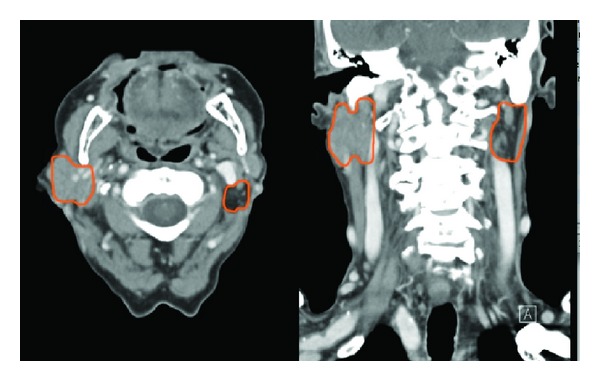
Head and neck CT scan (axial and coronal views) made 4 years after the left parotidectomy, showing a moderate partial atrophy of the autologous fat graft.

This 85 year-old male suffered a tumor at the inferior part of the left parotid gland, confirmed by a CT scan. A total parotidectomy with dissection and preservation of the facial nerve was performed because the tumor affected the deep part of the gland. Ten mL of fat was put in the surgical field (for 51 gr of tissue removed). Pathology showed a Warthin's tumor (or adenolymphoma). A slight facial paresis completely resolved 6 weeks later. Follow-up is 46 months. The cosmetic result is very good even there is a moderate atrophy of the fat graft and no complain of gustatory sweat syndrome is experienced by the patient. At one year, the Minor's iodine-scratch test is negative. A CT scan has been made 4 years after the procedure. While the cosmetic result and the symmetry are good, CT scan show a moderate atrophy of fat (Figures 3 and 4).

### 4.3. Case 3

**Figure 5:**
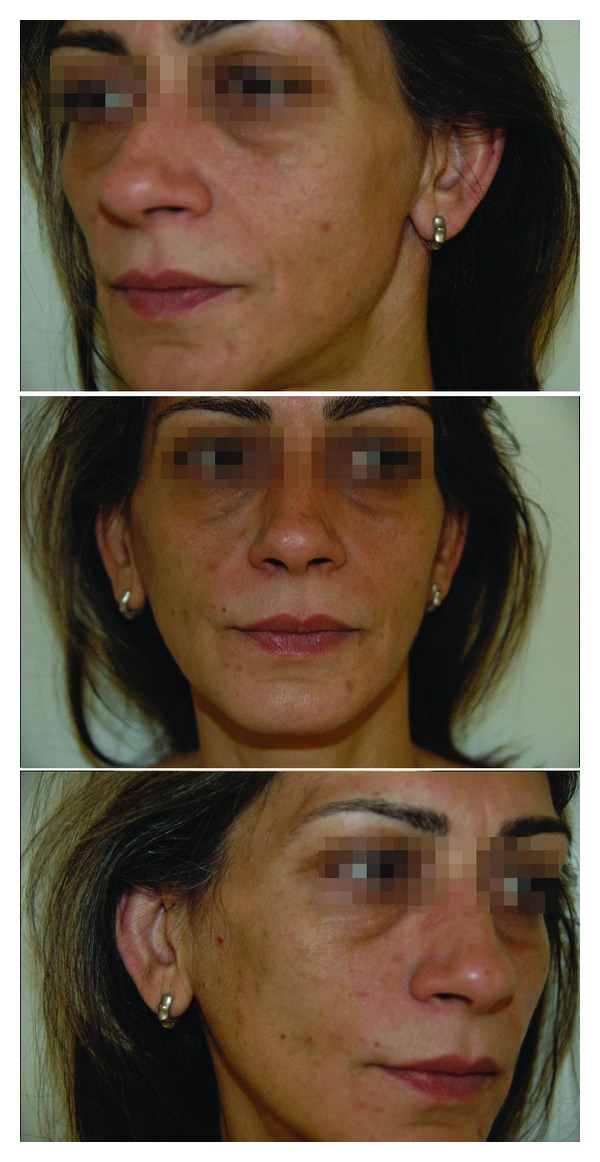
42 year-old woman 3 years after a superficial parotidectomy for a benign tumor of the parotid gland with immediate reconstruction by an autologous fat graft (13 mL). After that procedure, the result remained very good on the aesthetic point of view until a weight loss of 17 kg leading to a partial atrophy of the fat graft and to a clinical preauricular defect. There is no Frey's syndrome as objectivated by a Minor's starch-iodine test.

A 42 year-old woman underwent 3 years ago a superficial parotidectomy for a benign tumor of the parotid gland (Warthin's tumor) with immediate reconstruction by an autologous fat graft (13 mL). After that procedure, the result remained very good on the aesthetic point of view for 11 months, until a weight loss of 17 kg leading to a partial atrophy of the fat graft and subsequently to a clinical preauricular defect at the 19th postoperative month. But the result remains good and clearly better than without any immediate reconstruction (Figure 5). There is no clinical Frey's syndrome as demonstrated by a negative Minor's iodine-scratch test 19 months later.

Subjectively, all the patients were asymptomatic for Frey's syndrome. Minor's starch-iodine test is used to evaluate potential perspiration of the preauricular skin during mastication. This test consists of the application of an iodine-alcoholic solution on the concerned skin and when dried, application of starch. Then the patient masticates a chewing gum. The test is considered positive if the white powder of starch becomes black, traducing that skin perspirates. The Minor's starch-iodine test was performed 6 months to more than 1 year after the procedure for 8 patients and remains negative for all those patients.

The cosmetic results are subjectively considered by the patient and the surgeons as good to very good in all cases (mean postoperative follow-up of 21 months (range: 3–60)). A surprisingly slight to moderate atrophy of the fat graft is clinically observed in the long-term follow-up for all the patients, while more marked for two of the three irradiated patients (Table 1). This is contradiction with the current observed results we find in the medical literature. This could be explained by a peroperative overcorrection, an excellent local vascularization leading to a better survival of the fat graft or even a response to liberation of neurotransmitter by the cut axons of the traumatized facial nerve. We do not have any answer to that question; we are just able to observe and consider the clinical results. On another point of view, concerning the three cases of malignant tumors, the follow-up shows until now that fat graft does not impair the screening, on the clinical as well as on the radiological aspects.

## 5. Discussion

The Frey syndrome, also called gustatory sweat syndrome or auriculotemporal syndrome, is characterized by a sweating and a flushing and/or an erythema of the cheek area during mastication after parotid surgery [1]. It affects a minority of patients operated for a superficial or a total parotidectomy (following the series, from 2,6 to 14,3%) [2, 3].

It is caused by a regeneration of the cut secretomotor parasympathetic fibers between the facial nerve and the branches of the auriculotemporal or greater auricular nerves, responsible for an aberrant innervation of sweat glands and subcutaneous vessels, leading to the physiopathology of that syndrome. Like Jeong et al. who presented recently a paper on that subject [4], we feel that we should use an additional procedure primarily to avoid contour irregularity or deformity of the preauricular area (facial concavity leading to facial asymmetry) [5] as well as Frey's syndrome.

Many procedures are described to avoid or to treat the postoperative preauricular deformity and/or Frey's syndrome secondary to a parotidectomy; all of them are based on the principle of the interposition of a barrier between the facial nerve and the skin flap of the cheek. Considering autologous tissue, some authors used the sternocleidomastoid muscle flap [6, 7], a SMAS flap [8–10], and the temporoparietal fascial flap [11, 12]. Microvascular free flaps are also proposed, like rectus abdominis myocutaneous flap [13], lateral arm flap [14], gracilis and latissimus dorsi [15], deep inferior perforator flap, and anterolateral thigh flap [16]. Those methods can be associated with donor site morbidity, longer operative times, limited availability of tissue for larger defects, limited arc of rotation for local flaps, and inconsistent success rates in preventing Frey's syndrome [17].

Acellular human dermal matrix has also been used [17, 18], as well as lyophilized dura mater and expanded polytetrafluoroethylene soft tissue patch [19]. Nonautologous implants are associated with a higher risk of complications (infection, extrusion, and rejection) but are unlimited and readily available and useful to decrease significantly the incidence of Frey's syndrome [19].

Some authors used fat tissue: dermis-fat tissue [5, 20–22] or even free microvascular (dermis-) fat tissue transfer [23, 24].

Others use a two-stage surgical approach, with dermofat graft and lipofilling for the treatment of established Frey's syndrome and facial depression deformity, secondary to a previous parotidectomy [25].

We developed immediate reconstructive option after a case of secondary correction leading to a long lasting and permanent facial paresis. Secondary procedure can be useful in case of excessive fat resorption. We never did it because all the patients were satisfied with the aesthetic result.

Recently, Jeong et al. proposed to use the buccal fat pad [4]. Nonsurgical treatments are also proposed: botulinum toxin A, topical antiperspirants, antihistamine creams, anticholinergic agents, alcoholization of the otic ganglion, and tympanic neurectomy [26–29].

Autologous fat graft is an easy technique widely used in cosmetic surgery to correct folds of the face (nasolabial folds, etc.) and in reconstructive surgery to remodel soft tissue, to enlarge or to resurface them. This technique has many advantages: unlimited availability, no additional scarring, no donor site morbidity, little time consuming (especially in a two-team approach), and very good long-term results on the aesthetic as well as on the functional point of view. Coleman emphasizes the less traumatic technique possible for fat harvesting and placement, to preserve adipocytes. He insists on the fact that minimal amounts of fat should be transferred, in an intricate nest of tunnels in superimposed layers, maximizing the contact with surrounding tissues and the neovascularization of the adipocytes [30]. Concerning the technique we use, adaptations or modifications of Coleman's technique are to be highlighted: the cleansing technique and the injection technique.

Concerning the cleansing technique, Coleman advocates the centrifugation for many reasons such as removing nonviable fat aspirate components (oil, blood, and lidocaine) [31, 32]. However, many other authors find no advantage of centrifugation versus other methods; the technique is laborious and cumbersome with no better fat survival [33, 34]. In a recent paper, Botti et al. [35] demonstrated that there is no significant difference in terms of long-term results between the filtered and washed versus the centrifugated fat in cosmetic facial fat injections.

Moreover, if some authors report no difference in fat preparation techniques, others show increased graft survival rates after washing procedure [36, 37].

On the other hand, concerning the technique of injection, Coleman emphasizes the importance of the multilayered injection technique of a small amount of fat. In the case of a parotidectomy, this option does not exist anymore and we have no other option than to graft the fat* en bloc*. Following Coleman, this should lead to a very low survival of the graft. Our results show, on the contrary, a very good survival of the graft, leading to good or very good reproducible long-term results on the clinical (aesthetic as well as functional aspects) and on the radiological points of view. Even if there is a partial resorption of the fat graft, the clinical, functional, and radiological results are very encouraging.

## 6. Conclusion

Among the options to avoid complications of parotidectomy (Frey's syndrome and preauricular deformity), autologous fat transfer represents a very interesting option: unlimited availability of the fat tissue, no additional scarring, no donor site morbidity, not time consuming, very good to excellent, and reproducible long-term results on the aesthetic as well as the functional point of view. Our technique of *en bloc* use of the fat transfer in the operative field shows an unexpected very good survival of the graft. Even if this series is limited, the results are very encouraging and we think that autologous fat grafting has a place in the choice of alternative options.

## Figures and Tables

**Table 1:** Patients' data.

Age	Sex	Duration	Complication	gr	cc	FU	AP	Minor	RxΘ	Atrophy
59	F	2,75	None	18	10	60	Monomorph adenoma with foci of adenoid cystic carcinoma	0	Y	++
57	M	3,5	Transient VII paresis	40	20	12	Warthin	0		+
49	F	4	Transient VII paresis	30	15	23	Adenoid cystic carcinoma	0	Y	+
77	F	2,5	None	7	4	24	Oncocytic carcinoma	0	Y	++
65	F	3,5	Seroma	33	15	10	Pleomorphic adenoma	0		+
85	M	3	Transient VII paresis	51	10	46	Warthin	0		++
77	F	3	None	32	18	15	Pleomorphic adenoma	None		+
57	M	3,25	Transient VII paresis	29	16	3	Pleomorphic adenoma	0		+
42	F	2,5	Transient VII paresis; seroma	11	13	19	Warthin	0		++
58	M	3	Transient VII paresis; seroma	34	15	1	Warthin	None		?

Mean										
62	6 F; 4 M	3		28	14	21				

Duration: of the surgical procedure, in hours; gr: weight of the parotid specimen, in grams; cc: volume of fat grafted, in milliliters; FU: follow-up, in months; AP: pathology report; Minor: Minor's iodine-scratch test (0: negative; 1: positive; none: not performed); RxΘ: radiotherapy; Y: yes; atrophy: clinical fat atrophy; +: slight, ++: moderate, ?: unknown.
